# A Facile Synthesis of Arylazonicotinates for Dyeing Polyester Fabrics under Microwave Irradiation and Their Biological Activity Profiles

**DOI:** 10.3390/molecules171011495

**Published:** 2012-09-27

**Authors:** Saleh M. Al-Mousawi, Morsy A. El-Apasery, Huda M. Mahmoud

**Affiliations:** 1 Chemistry Department, Faculty of Science, Kuwait University, P.O. Box 5969, Safat 13060, Kuwait; 2 Dyeing, Printing and Textile Auxiliaries Department, Textile Research Division, National Research Centre, Dokki, Giza 12622, Egypt; 3 Department of Biological Sciences, Faculty of Science, Kuwait University, P.O. Box 5969, Safat 13060, Kuwait

**Keywords:** polyester fabrics, arylazonicotinates, disperse dyes, microwave irradiation, biological activity

## Abstract

A as textile dyes and the fastness properties of the dyed samples were measured. Most of the dyed fabrics tested displayed very good washing and perspiration fastness and series of 2-hydroxy- and 2-amino-6-substituted-5-arylazonicotinate monoazo compounds **7a**–**e** and **9a**–**c** were prepared via condensation of 3-oxo-3-substituted-2-arylhydrazonals **2a**–**e** with active methylene nitriles **3a**–**d** using microwave irradiation as an energy source. These substances were then tested moderate light fastness. Finally, the biological activity of the synthesized compounds against Gram positive bacteria, Gram negative bacteria and yeast were evaluated.

## 1. Introduction

Disperse dyes are very popular and are important class of dyes for dyeing polyester fabrics because of their brilliancy, wide range of hues, excellent fastness properties, besides environmental and economic reasons [1].

Disperse dyes are organic colors with less water solubility, so they are applied in colloidal aqueous dispersions to hydrophobic textile fibers in which the dyes literally dissolve and produce the desired coloration. The development of disperse dyes is due to significant increase in the World production of polyester fibers as compared to other fibers. Over 95% of disperse dyes usage is for the coloration of polyester and its blends. A monoazo dye with a heterocyclic system would be considered as a useful class of disperse dyes [2,3].

Derivatives of nicotinates, nicotinonitrile, and nicotinamide have a long history of use as heterocyclic components for various disperse dyes [4,5,6,7]. Moreover, they have been proven to constitute the active part of several biologically active compounds [8,9,10,11,12].

In view of these findings and in continuation of our previous studies [10,13] on the synthesis of a variety of 2-hydroxy- and 2-amino-6-substituted-5-arylazonicotinate compounds using traditional heating methods, in this article, a new synthesis of these compounds was synthesized under microwave irradiation and their application for dyeing polyester fabrics studied. In addition the biological activity of the synthesized dyes against *Escherichia coli* and *Pseudomonas aeruginosa*. (Gram negative bacteria), *Bacillus subtilis* and *Staphylococcus aureus* (Gram positive bacteria) and *Candida albicans* (yeast) was also studied.

## 2. Results and Discussion

### 2.1. Synthesis

Recently we have reported the synthesis of some 2-hydroxy- and 2-amino-6-substituted-5-arylazonicotinates dyes [13]. Herein, in an attempt to improve and facilitate the synthesis of these compounds, we report a new strategy for the preparation of these compounds in better yields by condensing of 3-oxo-3-substituted-2-arylhydrazonals with active methylene nitriles using microwave irradiation as an energy source.

**Scheme 1:**
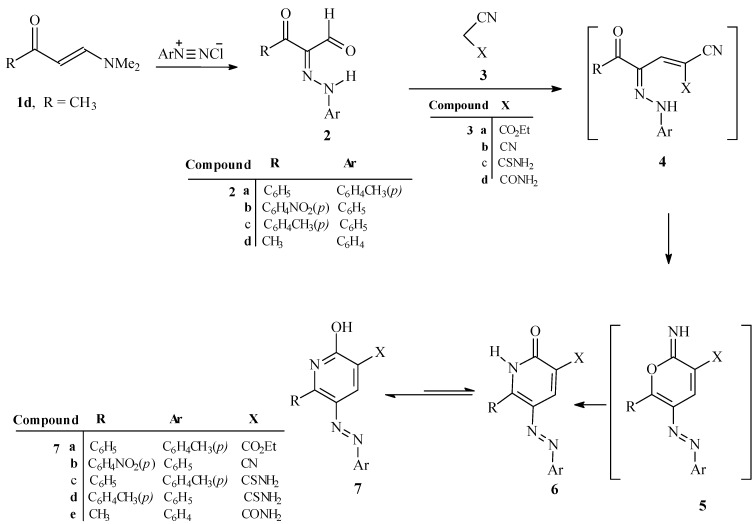
Synthesis of 2-hydroxy-6-substituted-5-arylazonicotinate derivatives **7a**–**e**.

We observed that reactions of **2a**–**d** with active methylenes **3a**–**d** in the presence of a catalytic amount of ammonium acetate and a few drops of acetic acid leads gives the corresponding 2-hydroxy-6-substituted-5-aryl azonicotinates **7a**–**e** (Scheme 1) after heating in a focused microwave oven at 170 °C for 1 min.

It is believed that the pathways for these processes involve initial reaction of **2a**–**d** with active methylenes **3a**–**d** to yield the hydrazono-enones **4** that then cyclize to generate the pyran-imines **5**. In the absence of ammonium ion, compounds **5** undergo a Dimroth type rearrangement to yield **7a**–**e**.

**Figure 1:**
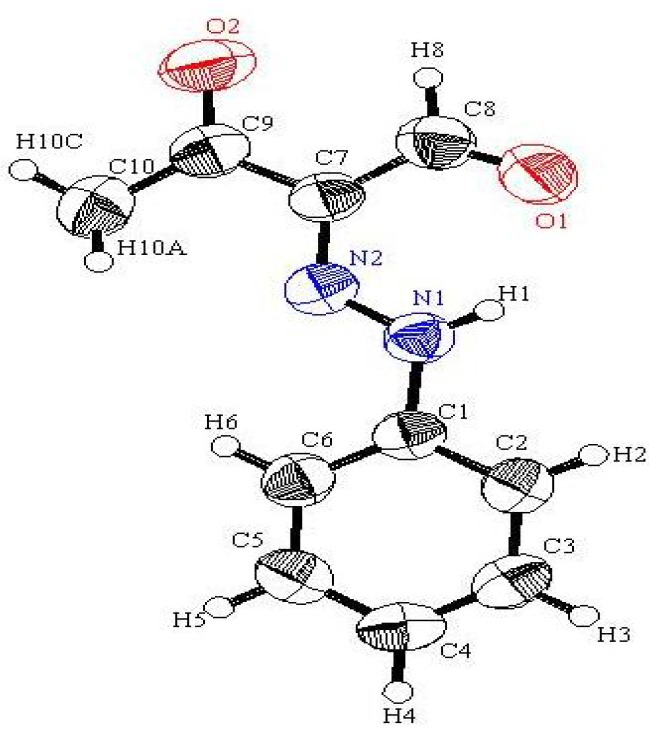
ORTEP drawing of **2d**.

Although not directly related to the processes described above, it is of value to note that earlier studies showed that hydrazono-3-oxo-propanal **2d** exists as an equilibrium mixture of *syn* and *anti*-forms [14] in dimethyl sulfoxide solution, with the latter stereoisomer being predominant. In contrast, the results of our current X-ray crystallographic studies demonstrate that in the solid state this substance only exists in the *anti*-form (Figure 1) [15].

**Scheme 2:**
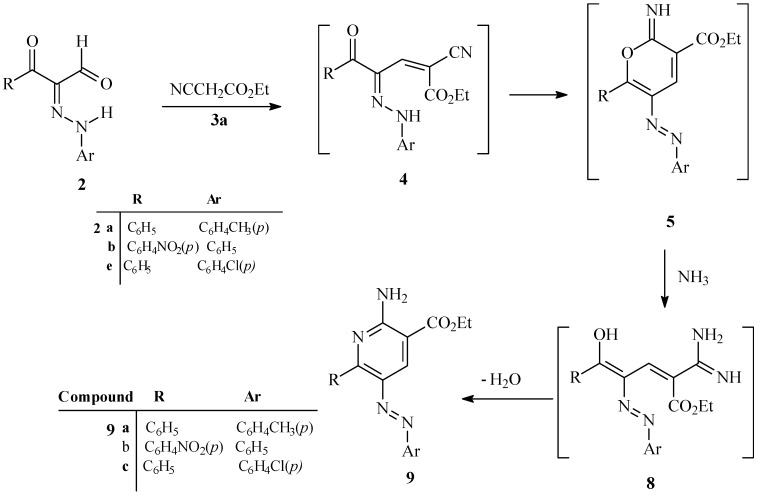
Synthesis of 2*-*amino-6-substituted-5-arylazonicotinate derivatives **9a**–**c**.

In contrast, when **2a,b,e** are condensed with ethyl cyanoacetate in the presence of excess ammonium acetate in a focused microwave oven at 170 °C for 1 min, ethyl 2-amino-6-substituted-5-arylazonicotinate derivatives **9a**–**c** are produced. It is believed that the pathways for these processes involve initial reaction of **2a,b,e** with ethyl cyanoacetate to yield the hydrazono-enones **4** that then cyclize to generate the pyran-imines **5**. In the presence of a high concentration of ammonium acetate, pyran-imines **5** participate undergo a ring opening to yield amidines **8** that then cyclize followed by water elimination to yield **9a**–**c** (Scheme 2).

### 2.2. Dyeing

The functionalized compounds **7a**–**e** and **9a**–**c** were applied to polyester fabrics at 3% weight of fibre using the high temperature dyeing method at 130 °C. Color shades varying from yellow to brownish-green were obtained. The dyeing on the polyester fabrics was evaluated in terms of their fastness properties (e.g., fastnesses to washing, perspiration, and light). The optical measurements and fastness property data for the dyed fabrics are listed in Table 1 and Table 2. The color of dyeing on polyester fabrics is expressed in terms of CIELAB values (Table 1), and the following CIELAB coordinates were measured: lightness (L *); chroma (C *); hue angle (*H*) from 0 to 360°; A *, whose value represents the degree of redness (positive) and greenness (negative); and B *, whose value represents the degree of yellowness (positive) and blueness (negative). A reflectance spectrophotometer was used for the colorimetric measurements of the dyed samples. The *K/S* values (where *K* is the absorbance coefficient and *S* is the scattering coefficient) given by the reflectance spectrometer were calculated at *λ*_max_ (wavelength of maximum absorption) and were directly correlated with the dye concentration on the dye substrate according to the Kubelka–Munk equation. In general, the color hues of the arylazonicotinates **7b** on the polyester fabric shifted to the greenish directions; this was indicated by the low value of A * = 0.87 (red–green axis). The positive values of B * (yellow-blue axis) indicated that the color hues of the arylazonicotinates **7a,d** and **9a**–**c** on the polyester fabric shifted in the yellowish direction.

**Table 1:** Optical measurements of the synthesized monoazo compounds on the polyester fabrics.

Dye No.	UV, [ *λ*_max_ (DMF)/nm]	Molar extinction coefficient (mol^−1^ cm ^−1^)	*K/S*	L *	A *	B *	C *	*H*
**7a**	371	16,484	19.30	51.58	14.63	39.13	41.78	69.50
**7b**	368	9,237	20.48	34.08	0.87	12.74	12.77	86.11
**7c**	349	6,813	17.10	40.76	9.15	25.31	26.92	70.13
**7d**	347	11,762	16.89	58.82	8.74	36.37	37.41	76.49
**7e**	360	10,769	10.27	50.60	4.65	22.63	23.10	78.40
**9a**	374	16,524	22.02	54.95	18.01	48.60	51.83	69.67
**9b**	373	9,499	21.22	52.53	13.85	41.29	43.56	71.44
**9c**	379	25,924	25.50	56.06	21.78	57.05	61.07	69.10

The physical data for the dyed fabrics, given in Table 2, shows that these compounds displayed very good washing and perspiration fastness. The light fastness is moderate with respect to most of the tested compounds, except for **7d** and **9b** which showed good results.

**Table 2:** Fastness properties of monoazo compounds on polyester fabrics*.*

Dye No.	Color shade on polyester	Wash fastness			Perspiration fastness						Light fastness
					Alkaline			Acidic			
		Alt	SC	SW	Alt	SC	SW	Alt	SC	SW	
**7a**	Yellow	5	5	5	5	5	5	5	4–5	5	2–3
**7b**	Brownish-green	5	5	5	5	5	5	5	4–5	5	2–3
**7c**	Yellowish-brown	4–5	4–5	4–5	5	5	5	5	4–5	5	2–3
**7d**	Yellow	5	4–5	5	5	5	5	5	4–5	5	4–5
**7e**	Yellowish-brown	5	5	5	5	5	5	5	4–5	5	3–4
**9a**	Dark yellow	5	4–5	5	5	5	5	5	4–5	5	2–3
**9b**	Yellowish-brown	5	5	5	5	5	5	5	4–5	5	4–5
**9c**	Yellowish-orange	5	5	5	5	5	5	5	4–5	5	3–4

### 2.3. Antimicrobial Activities

The antimicrobial activities of the synthesized compounds were screened against selected bacteria and fungi by the agar well diffusion method and their inhibition zones diameters, given in Table 3, reveal that all of the tested arylazonicotinates compounds showed positive antimicrobial activities against at least one of the tested microorganisms. All of them showed strong activities (>10 mm inhibition zone) against *S. auerus*. Two of the compounds **7a** and **9a**, showed medium activities against Gram negative bacteria. Where most of the compounds showed no activities against the two strains of Gram negative bacteria used in the study. Also the majority of compounds showed weak to no activities at all with *B. subtilis.* Only dye **7d** showed significant inhibition zone > 10 mm, against *Candida albican*. The other compound that showed medium activities against yeast is **7c** while all the other compounds failed to affect the yeast growth.

**Table 3:** Diameter of the zones of inhibition of the tested compounds that showed weak to strong antimicrobial against microorganisms.

Compound No	Inhibition zone diameter (Nearest mm)				
	*B. subtilis* Mean ± SD	*S. aureus* Mean ± SD	*E. coli* Mean ± SD	*P. aeruginosa* Mean ± SD	*C. albicans* Mean ± SD
**7a**	0.1 ± 0.08	15 ± 0.02	7 ± 0	-	-
**7b**	-	11 ± 0.87	-	-	-
**7c**	0.7 ± 0.13	16 ± 0	-	-	7 ± 0.07
**7d**	0.1 ± 0	16 ± 0.08	-	-	12 ± 0.1
**9a**	-	13 ± 0	7 ± 0	7 ± 0.05	-
**9b**	-	10 ± 0.2	-	-	-
**Ampicillin** *	30 ± 0.05	46 ± 0.7	31 ± 0.14	17 ± 0.07	
**Cyloheximide** **					-

(-) no inhibition; * Ampicillin: antibacterial (100 mg·mL^−1^); ** Cycloheximide: antifungal (100 mg·mL^−1^), SD = Standard Deviation.

**Figure 2:**
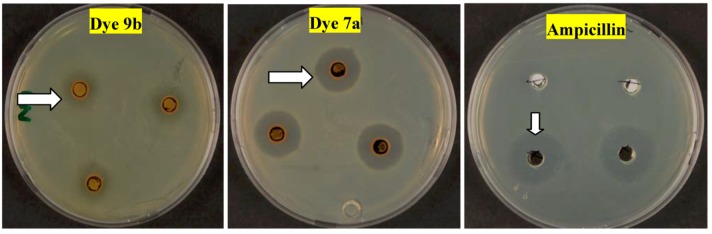
*Staphylococcus aureus* treated with 10 mg·mL^−1^ of compounds **9b** and **7a** with comparison to Ampicillin after six days of incubation.

It is of value to mention here that as Figure 2 shows, after six days the inhibition zone did not show any difference in the size, yet the zone is not clear which indicates that the compound number **9b** did not kill the microorganisms, but rather had weakened their growth only, this is comparison to compound number **7a** or to ampicillin as reference.

Therefore, the new synthesized classes showed promising results regarding the potential to be utilized for medicinal purposes. Currently, we are inspecting the biological activity of the polyester fabrics dyed with the arylazonicotinate compounds against Gram positive bacteria, Gram negative bacteria and yeast.

## 3. Experimental

### 3.1. General

Melting points were recorded on a Gallenkamp apparatus. IR spectra were recorded using KBr pellets on a JASCO FTIR-6300 FT-IR spectrophotometer. ^1^H- and ^13^C-NMR spectra were recorded on Bruker DPX 400 MHz or AvanceII 600 MHz super-conducting NMR spectrometers with proton spectra measured at 400, 600 MHz and carbon spectra at 100 and 150 MHz, respectively. Mass spectra were measured on a high resolution GC/MS DFS-Thermo. Microanalyses were performed on Elementar-Vario Micro cube Analyzer. X-Ray analyses were performed using a Rigaku Rapid II diffractometer. The microwave oven used is a single mode cavity Explorer Microwave (CEM Corporation, Matthews, NC, USA). The dyeing of polyester fabrics were conducted using LINITEST + Laboratory High Temperature Dyeing and Fastness System (ATLAS MTT GmbH, Vogelbergstrasse 22, Altenhasslau, Germany). The colorimetric parameters of the dyed polyester fabrics were determined on a reflectance spectrophotometer (UltraScan PRO D65, HunterLab, Virginia, VA, USA).

*Synthesis of 3-oxo-2-(phenylhydrazono)-butyraldehyde* (**2d**). A cold solution of the diazonium salt (10 mmol), prepared by adding a cold solution of sodium nitrite (0.7 g) in water (5 mL) to a solution of the corresponding aniline (10 mmol) in conc. HCl (5 mL), was added to a cold solution of enaminone **1d** (10 mmol) in EtOH (10 mL) containing NaOH (1.6 g). The resulting mixture was stirred at room temperature for 1 h. The precipitate that formed was collected by using filtration and crystallized from EtOH to give **2d**. This compound was obtained as buff crystals (82%); mp. 124 °C (Lit. 122 °C) [16]; IR (KBr): = 3137 (NH), 1669 (CO) cm^−1^; ^1^H-NMR (DMSO-*d_6_*): δ = 2.50 (s, H, CH_3_), 7.25 (t, 1H, *J* = 7.2 Hz phenyl-H), 7.46 (t, 2H, *J* = 8.4 Hz phenyl-H), 7.64 (d, 2H, *J* = 8.4 Hz phenyl-H); 9.93 (s, 1H, CHO, D_2_O exchangeable), 14.14 (s, 1H, NH, D_2_O exchangeable).^13^C-NMR (DMSO-*d6*): δ = 196.3 (CO), 189.7 (CHO), 141.3, 133.5, 129.6, 126.2, 116.8, 24.3 (CH_3_); MS, *m/z* (%), 190 (M^+^, 100), 148.1 (80), 120.1 (58), 77.0 (71). HRMS: *m/z* (EI) for C_10_H_10_N_2_O_2_; calcd. 190.0736; Found: 190.0736.

General Procedure for the Preparation of Compounds **7a**–**e**

*Method A*: Independent mixtures of **2a**–**e** (0.01 mol), active methylene nitrile derivatives **3a**–**d** (0.01 mol), and ammonium acetate (0.5 g) in acetic acid (2 mL) were irradiated by focused microwaves at 170 °C for 1 min. Completion of the reactions was monitored by TLC. The pressure build-up in the closed reaction vessel was carefully monitored. After the irradiation, the reaction tube was cooled with high-pressure air through an inbuilt system in the instrument until the temperature had fallen below 50 °C. The mixtures were cooled and then poured into ice-water. The solids that formed were collected by using filtration and crystallized from proper solvents to give **7a**–**e**.

*2-Hydroxy-6-phenyl-5-p-tolylazonicotinic acid ethyl ester* (**7a**). This compound was obtained as yellowish-brown crystals (84%); mp. 180–182 °C; IR (KBr): = 3401 (OH), 1692 (CO) cm^−1^; ^1^H-NMR (DMSO-*d_6_*): δ = 1.35 (t, 3H, *J* = 7.2 CH_3_), 2.50 (s, 3H, CH_3_), 4.36 (q, 2H, *J* = 7.2 CH_2_), 7.32 (d, 2H, *J* = 8.0 Hz arom-H), 7.48–7.50 (m, 3H, arom-H), 7.60 (d, 2H, *J* = 8.0 Hz arom-H), 7.78–7.79 (m, 2H, arom-H), 7.98 (s, 1H, OH, D_2_O exchangeable). 8.54 (s, 1H, arom-H); ^13^C-NMR (DMSO-*d_6_*): δ = 166.5, 161.0, 158.5, 154.5, 150.6, 142.9, 138.3, 136.5, 131.0, 130.8, 129.8, 127.4, 122.3, 105.2, 61.0, 20.9, 14.1; MS, *m/z* (%), 361 ([M]^+^, 54), 314 (25), 289 (44), 213 (6), 182 (7), 105 (100), 91 (42), 77 (48). *λ*_max_ (DMF)/nm 371. HRMS: *m/z* (EI) for C_21_H_19_N_3_O_3_; calcd. 361.1418; Found: 361.1418*.*

*2-Hydroxy-6-(4-nitrophenyl)-5-phenylazonicotinonitrile* (**7b**). This compound was obtained as brownish-green powder (85%); mp. 276–278 °C; IR (KBr): = 3446 (OH), 2201 (CN) cm^−1^; ^1^H-NMR (DMSO-*d_6_*): 7.25–7.62 (m, 6H, arom-H, OH), δ = 7.27 (d, 1H, *J* = 8.8 Hz arom-H), 8.01–8.08 (m, 1H, arom-H), 8.20 (d, 1H, *J* = 8.8 Hz arom-H), 8.27–8.36 (m, 2H, arom-H), ^13^C-NMR (DMSO-*d_6_*): δ = 159.8, 152.2, 151.1, 147.6, 141.7, 139.6, 139.0, 131.1, 129.7, 129.2, 126.3, 123.4, 123.1, 114.0, MS, *m/z* (%), 345 ([M]^+^, 6), 268 (10), 239 (100), 210 (12), 194 (42), 150 (10), 93 (10), 57 (20). *λ*_max _(DMF)/nm 368. Anal. Calcd for C_18_H_11_N_5_O_3_: C, 62.61; H, 3.21; N, 20.28. Found: C, 62.78; H, 3.28; N, 20.20.

*2-Hydroxy-6-phenyl-5-p-tolylazothionicotinamide* (**7c**). This compound was obtained as brown powder (84%); mp. 276–278 °C; IR (KBr): = 3441 (OH), 3385, 3291 (NH_2_), 1660 (CO) cm^−1^; ^1^H-NMR (DMSO-*d_6_*): δ = 2.26 (s, 3H, CH_3_), 7.02–8.01 (m, 9H, arom-H), 8.57 (s, 1H, OH), 8.76 (s, 1H, pyridyl-H), 12.38 (s, 2H, NH_2_). ^13^C-NMR (DMSO-*d_6_*): δ = 206.0, 162.2, 158.0, 152.7, 141.7, 138.8, 137.3, 135.8, 131.7, 130.6, 129.4, 129.0, 124.5, 94.0, 20.8; MS, *m/z* (%), 348 ([M]^+^, 5), 315 (100), 300 (28), 279 (8). *λ*_max_ (DMF)/nm 349. Anal. Calcd for C_19_H_16_N_4_OS: C, 65.50; H, 4.63; N, 16.08; S, 9.20. Found: C, 65.60; H, 4.30; N, 16.43; S, 8.83.

*2-Hydroxy-5-phenylazo-6-p-tolylthionicotinamide* (**7d**). This compound was obtained as yellowish-brown powder (72%); mp. 166–168 °C; IR (KBr): = 3409 (OH), 3283, 3197 (NH_2_), 1697 (CO) cm^−1^; ^1^H-NMR (DMSO-*d_6_*): δ = 2.33 (s, 3H, CH_3_), 7.33–7.95 (m, 9H, arom-H), 8.32 (s, 1H, OH), 8.77 (s, 1H, pyridyl-H), 10.33 (s, 1H, NH), 10.64 (s, 1H, NH). ^13^C-NMR (DMSO-*d_6_*): δ = 206.5, 162.1, 158.1, 144.4, 141.1, 137.3, 133.2, 132.3, 130.7, 129.2, 129.0, 128.8, 125.6, 115.2, 21.2; MS, *m/z* (%), 348 ([M]^+^, 5), 315 (100), 300 (32), 290 (6). *λ*_max_ (DMF)/nm 347. Anal. Calcd for C_19_H_16_N_4_OS: C, 65.50; H, 4.63; N, 16.08; S, 9.20. Found: C, 65.78; H, 4.39; N, 16.43; S, 9.36.

*2-Hydroxy-6-methyl-5-phenylazonicotinamide* (**7e**). This compound was obtained as brown powder (74%); mp. >300 °C; IR (KBr): = 3446 (OH), 3342, 3242 (NH_2_), 1657 (CO) cm^−1^; ^1^H-NMR (DMSO-*d_6_*): δ = 2.39 (s, 3H, CH_3_), 7.56–7.69 (m, 5H, phenyl-H), 7.95 (s, 1H, OH, D_2_O exchangeable), 8.25 (s, 1H, pyridyl-H), 11.30 (s, 2H, NH_2_, D_2_O exchangeable). ^13^C-NMR (DMSO-*d_6_*): δ = 168.1, 153.9, 149.0, 139.5, 130.8, 130.4, 128.8, 126.7, 125.0, 97.3, 15.7; MS, *m/z* (%), 256 ([M]^+^, 12), 207 (17), 129 (32), 93 (54), 77 (100). *λ*_max_ (DMF)/nm 360. Anal. Calcd for C_13_H_12_N_4_O_2_: C, 60.93; H, 4.72; N, 21.86. Found: C, 61.43; H, 4.06; N, 21.81.

*Method B:* Independent mixtures of **2a**–**e** (0.01 mol), active methylene nitrile derivatives **3a**–**d** (0.01 mol), and ammonium acetate (0.5 g) in acetic acid (10 mL) were stirred at reflux for 30 min. Completion of the reactions was monitored by TLC. The same treatment as described in method A to give **7a**–**e** in (83, 91, 77 [13]), 68 and 70%, respectively.

General Procedure for the Preparation of Compounds **9a**–**c**

*Method A*: Independent mixtures of **2a**,**b** or **2e** (0.01 mol), ethyl cyanoacetate (0.01 mol), and ammonium acetate (2 g) in acetic acid (2 mL) were irradiated by focused microwaves at 170 °C for 1 min. Completion of the reactions was monitored by TLC. The mixtures were cooled and then poured into ice-water. The solids that formed were collected by using filtration and crystallized from proper solvents to give **9a**–**c**.

*2-Amino-6-phenyl-5-p-tolylazonicotinic acid ethyl ester* (**9a**). This compound was obtained as brownish-yellow crystals (81%); mp. 210–212 °C; IR (KBr): = 3402 , 3275 (NH_2_) 1693 (CO) cm^−1^; ^1^H-NMR (DMSO-*d_6_*): δ = 1.37 (t, 3H, *J* = 7.2 CH_3_), 2.50 (s, 3H, CH_3_), 4.39 (q, 2H, *J* = 7.2 CH_2_), 7.31 (d, 2H, *J* = 8.4 Hz arom-H), 7.47–7.48 (m, 3H, arom-H), 7.98 (s, 2H, NH_2_, D_2_O exchangeable).7.59 (d, 2H, *J* = 8.4 Hz arom-H), 7.80–7.82 (m, 2H, arom-H), 8.54 (s, 1H, arom-H); ^13^C-NMR (DMSO-*d_6_*): δ = 166.2, 161.3, 159.5, 150.4, 140.6, 137.2, 136.5, 130.8, 129.8, 129.2, 127.4, 127.2, 122.3, 104.9, 61.0, 20.9, 14.1; *λ*_max_ (DMF)/nm 374. Anal. Calcd for C_21_H_20_N_4_O_2_: C, 69.98; H, 5.59; N, 15.55. Found: C, 69.99; H, 5.50; N, 15.25*.* HRMS: *m/z* (EI) for C_21_H_20_N_4_O_2_; calcd. 360.1582; Found: 360.1582.

*2-Amino-6-(4-nitrophenyl)-5-phenylazonicotinic acid ethyl ester* (**9b**). This compound was obtained as yellowish-brown powder (80%); mp. 200–202 °C; IR (KBr): = 3385, 3201 (NH_2_), 1656 (CO) cm^−1^; ^1^H-NMR (DMSO-*d_6_*): δ = 1.32 (t, 3H, *J* = 7.2 CH_3_), 4.35 (q, 2H, *J* = 7.2 CH_2_), 7.32–7.62 (m, 3H, arom-H), 7.68 (d, 2H, *J* = 7.2 Hz arom-H), 8.02 (d, 2H, *J* = 8.4 Hz arom-H), 8.16–8.41 (m, 4H, arom-H, NH_2_), 8.56 (s, 1H, pyridyl-H); ^13^C-NMR (DMSO-*d_6_*): δ = 166.2, 163.2, 159.8, 152.3, 148.6, 143.7, 136.8, 132.3, 131.4, 130.7, 128.6, 124.1, 122.7, 106.3, 61.0, 14.2; MS, *m/z* (%), 391 ([M]^+^, 100), 360 (28), 286 (22), 256 (64), 213 (13), 152 (12), 111 (21), 97 (42), 77 (72). *λ*_max_ (DMF)/nm 373. HRMS: *m/z* (EI) for C_20_H_17_N_5_O_4_; calcd. 391.1275; Found: 391.1275.

*2-Amino-5-(4-chlorophenylazo)-6-phenylnicotinic acid ethyl ester* (**9c**). This compound was obtained as yellowish-orange powder (68%); mp. 188–190 °C; IR (KBr): = 3275 , 3182 (NH_2_), 1693 (CO) cm^−1^; ^1^H-NMR (DMSO-*d_6_*): δ = 1.36 (t, 3H, *J* = 7.2 CH_3_), 4.36 (q, 2H, *J* = 7.8 CH_2_), 7.23 (d, 1H, *J* = 10.2 Hz arom-H), 7.39 (d, 1H, *J* = 7.8 Hz arom-H), 7.49–7.82 (m, 7H, arom-H, NH_2_), 7..89 ((d, 1H, *J* = 10.2 Hz arom-H), 7.98 (d, 1H, *J* = 7.8 Hz arom-H), 8.56 (s, 1H, pyridyl-H); ^13^C-NMR (DMSO-*d_6_*): δ = 166.1, 161.9, 159.7, 150.9, 141.7, 139.8, 137.1, 136.4, 134.8, 129.8, 127.3, 123.9, 105.0, 61.0, 14.1; *λ*_max_ (DMF)/nm 379. Anal. Calcd for C_20_H_17_ClN_4_O_2_: C, 63.08; H, 4.50; N, 14.71. Found: 63.55; H, 4.37; N, 14.77. HRMS: *m/z* (EI) for C_20_H_17_ClN_4_O_2_; calcd. 380.1035; Found: 380.1035.

*Method B*: Independent mixtures of **2a,b** or **2e** (0.01 mol), ethyl cyanoacetate (0.01 mol), and ammonium acetate (3 g) in acetic acid (10 mL) were stirred at reflux for 30 min. Completion of the reactions was monitored by TLC. The same treatment as described in method A gave **9a**–**c** in 83 [13], 60, and 65%, respectively.

### 3.2. High Temperature Dyeing Method *(HT)*

#### 3.2.1. Materials

Scoured and bleached polyester 100% (150 130 g/m^2^, 70/2 denier) was obtained from El-Shourbagy Co., Egypt. The fabric was treated before dyeing with a solution containing non-ionic detergent (Sera Wash M-RK, 5 g/L) and sodium carbonate (2 g/L) in a ratio of 50:1 at 60 °C for 30 min, then thoroughly washed with water and air dried at room temperature.

#### 3.2.2. Dyeing

A dispersion of the dye was produced by dissolving the appropriate amount of dye (3% shade w.o.f) in acetone (1 mL) and then added dropwise with stirring to the dyebath (liquor ratio 50:1) in the presence of a 1:1 ratio of Sera Gal P-LP as dispersing agent. The pH value of the bath was adjusted to 4.5–5 with acetic acid (10%) in the presence of a 1:1 ratio of the dispersing agent (Sera Gal P-LP). The temperature was raised to 130 °C at the rate of 7 °C/min, and dyeing continued for 60 min. After dyeing, the fabrics were thoroughly washed and then subjected to a surface reduction cleaning [(2 g NaOH + 2 g sodium hydrosulphite)/L]. The samples were heated in this solution for 30 min. at 70 °C and then thoroughly washed and air-dried.

### 3.3. Color Measurements and Analyses

#### 3.3.1. Color Measurements

The colorimetric parameters (Table 1) of the dyed polyester fabrics were determined on a reflectance spectrophotometer (UltraScan PRO D65). The color yields of the dyed samples were determined by using the light reflectance technique performed on a Perkin-Elmer (Lambda 3B) UV/VIS Spectrophotometer. The color strengths, expressed as K/S values, were determined by applying the Kubelka-Mink equation as follows: 


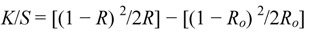


where *R* = decimal fraction of the reflectance of the dyed fabric; *R_o_* = decimal fraction of the reflectance of the undyed fabric; *K* = absorption coefficient; *S* = scattering coefficient.

#### 3.3.2. Fastness Testing

Fastnesses to washing, perspiration, and light were tested according to reported methods [17].

### 3.4. Antimicrobial Activities Test

The antimicrobial activities of different arylazonicotinates compounds were tested using the agar-well diffusion technique (Isaacson and Kirchbaum, [18]) against five different microbial cultures. Pure cultures of *Escherichia coli* and *pseudomonas aeruginosa* (Gram negative bacteria), *Bacillus subtilis* and *Staphylococcus aureus* (Gram positive bacteria) and *Candida albicans* (yeast) were involved in the test. An aliquot of each bacterial strain (0.1 mL) was inoculated and spread on nutrient agar (NA) while the yeast (0.1 mL) was spread on potato dextrose agar (PDA). The inoculated plates were supplied with 100 µL of each of the tested compounds with a total final concentration of 10 mg mL^−1^. The compounds were included in 4 mm wells produced by sterile cork borer. The NA plates were incubated at 37 °C for 24 h while PDA plates were incubated at 25 °C for 24–48 h. The zones of inhibition around the wells were determined and the average based on three replica was recorded. cycloheximide and ampicillin, both used as references in the experiment as cycloheximide is known to inhibit eukaryotic organisms while ampicillin inhibits prokaryote organisms. All plates were kept for six days after inoculation and the changes in the inhibition zone was monitored and documented by photography in order to determine on the cytolytic and cytostatic effect of the tested compounds.

## 4. Conclusions

A series of arylazonicotinates derivatives were synthesized via condensation of 3-oxo-3-substituted-2-arylhydrazonals with active methylenes using microwave irradiation as an energy source. The new compounds were tested as textile dyes on polyester fabrics, where they display yellow to brownish-green hues, in addition to very good washing and perspiration fastness and moderate light fastness. Finally, the biological activity of the synthesized compounds against Gram positive bacteria, Gram negative bacteria and yeast was determined.
